# Schnelle Abheilung einer schweren Psoriasis vulgaris bei einem HIV-positiven Patienten unter antiretroviraler Therapie

**DOI:** 10.1007/s00105-023-05107-8

**Published:** 2023-01-31

**Authors:** L. Bauditz, N. Booken, S. W. Schneider, F. Abeck

**Affiliations:** Klinik und Poliklinik für Dermatologie und Venerologie, Universitätsklinikum Hamburg-Eppendorf, Martinistr. 52, 20246 Hamburg, Deutschland

**Keywords:** Psoriasis, HIV, Antiretrovirale Therapie, Psoriasis-Therapie, Komorbiditäten, Psoriasis, Human immunodeficiency virus, Antiretroviral therapy, Psoriasis treatment, Comorbidities

## Abstract

Im Rahmen einer HIV-Erkrankung kann es zur Exazerbation einer Psoriasis vulgaris kommen. Wir präsentieren den Fall eines Patienten mit schwerer Psoriasis vulgaris (PASI 34,2) und HIV-Erstdiagnose, der unter der Einleitung einer antiretroviralen Therapie eine nahezu vollständige Abheilung der Psoriasis zeigte. Die antiretrovirale Therapie stellt eine effektive Behandlungsoption der HIV-assoziierten Psoriasis dar. Insbesondere im Falle einer plötzlichen Exazerbation oder Therapieresistenz der Psoriasis sollte die Durchführung einer HIV-Diagnostik in Erwägung gezogen werden, da das Wissen über eine zugrunde liegende Infektion elementar für die Wahl des Therapieansatzes ist.

## Abstract (en)

Psoriasis exacerbation may occur in patients with human immunodeficiency virus (HIV). We present the case of a patient with severe psoriasis vulgaris (PASI 34.2) and an initial HIV diagnosis who showed rapid resolution of psoriasis upon initiation of antiretroviral therapy. Antiretroviral therapy is an effective treatment option for HIV-associated psoriasis. Especially in patients with a sudden psoriasis flare-up or resistance to therapy, HIV testing should be considered as knowledge of an underlying infection is elementary to treatment decision-making.

## Anamnese

Wir berichten über einen 57-jährigen Patienten, der sich aufgrund einer schweren Exazerbation einer seit 2016 bekannten Psoriasis vulgaris in unserer Klinik vorstellte. Die Psoriasis war in den letzten Jahren unter topischer Behandlung stets gut kontrolliert gewesen. Eine Licht- oder Systemtherapie war bisher nicht erfolgt. Ein Auslöser für die Exazerbation konnte anamnestisch nicht identifiziert werden, jedoch gab der Patient eine verminderte Leistungsfähigkeit an. Eine B‑Symptomatik wurde verneint. Die Familienanamnese für eine Psoriasis war positiv. Weitere Vorerkrankungen bestanden mit Ausnahme eines Nikotinabusus nicht.

## Befund

**Figure 1:**
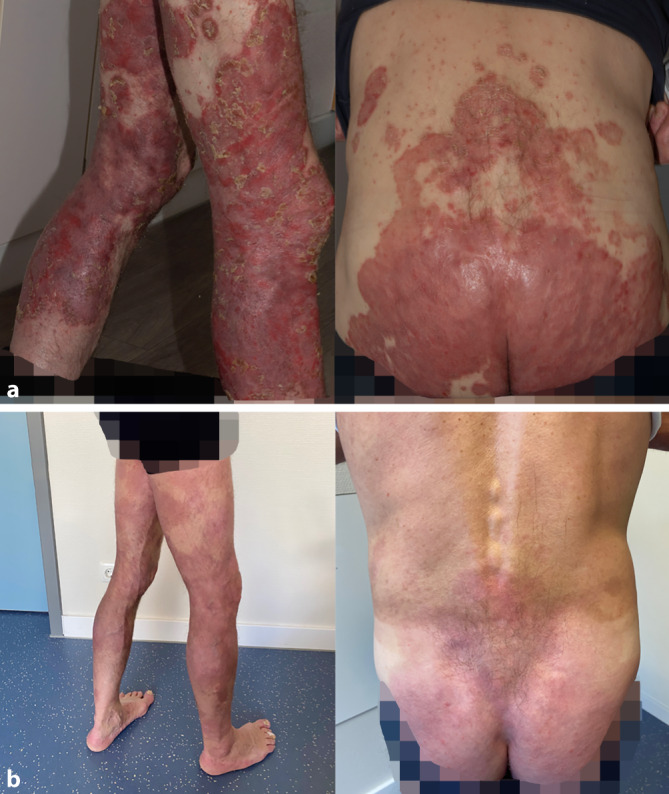

Am gesamten Integument zeigten sich unter Betonung der Extremitäten und der Genital- und Glutealregion stark infiltrierte, scharf begrenzte erythematosquamöse Plaques (Abb. 1a). Die Nägel der Hände und Füße imponierten dystroph mit subungualen Hyperkeratosen und Tüpfelnägeln. Das Gaenslen-Zeichen war beidseits negativ. Anzeichen einer Daktylitis, einer Palmar- oder Plantarfasziitis oder Enthesitis waren nicht ersichtlich. Die weitere Ganzkörperuntersuchung zeigte keine Auffälligkeiten, insbesondere keine Lymphadenopathie.

## Diagnostik

Bei einem Psoriasis Area Severity Index (PASI) von 34,2 lag eine schwere Psoriasis vulgaris vor, einhergehend mit einer schweren Beeinträchtigung der Lebensqualität (Dermatologischer-Lebensqualitäts-Index [DLQI] von 21). Es erfolgten laborchemische Voruntersuchungen vor Einleitung einer Systemtherapie. Nach Zustimmung des Patienten erfolgte außerdem eine serologische Testung auf das humane Immundefizienzvirus (HIV). Hierdurch ließen sich eine zuvor nicht bekannte HIV-Infektion mit einer Viruslast von 255.000 Kopien/ml sowie eine deutlich erniedrigte Zahl an CD4^+^-Lymphozyten von 192/µl (Referenz 500–1350/µl) nachweisen (Stadium CDC [Center for Disease Control] A3).

## Therapie und Verlauf

Aufgrund der HIV-Erstdiagnose erfolgte eine infektiologische Mitbeurteilung. Es wurden eine kombinierte antiretrovirale Therapie mit Bictegravir/Emtricitabin/Tenofoviralafenamid sowie eine Prophylaxe opportunistischer Infektionen mit Cotrimoxazol eingeleitet. Zusätzlich wurden weitere sexuell übertragbare Infektionen laborchemisch ausgeschlossen (Syphilis, Hepatitis B, Gonokokken und Chlamydien).

**Figure 2:**
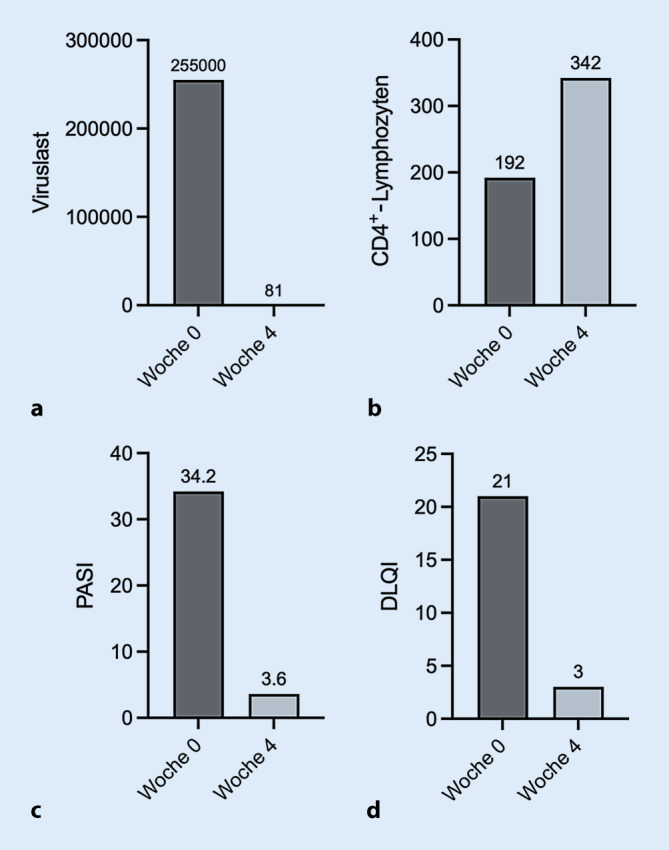

Es erfolgten eine intensivierte Lokaltherapie bestehend aus Steroid- und Vitamin-D-haltigen Externa sowie die Einleitung einer Lichttherapie mittels Bade-PUVA (5 Bestrahlungen, Kumulativdosis 2,4 J/cm^2^), die nach Eingang der HIV-Diagnose auf UVB-311 nm (8 Bestrahlungen, Kumulativdosis 3,6 J/cm^2^) umgestellt wurde. Bei Exazerbation der Psoriasis im zeitlichen Zusammenhang mit der HIV-Erstdiagnose als identifizierbarer Auslöser wurde die Einleitung einer Systemtherapie der Psoriasis vorerst zurückgestellt. Vier Wochen nach Einleitung der antiretroviralen Therapie ließ sich ein Rückgang der HIV-Viruslast von 255.000 auf 88 Kopien/ml (Abb. 2a) sowie ein Anstieg der CD4^+^-Lymphozyten von 192 auf 300/µl verzeichnen (Abb. 2b). Die Prophylaxe mit Cotrimoxazol konnte abgesetzt werden. Unter der genannten Therapie zeigte sich eine rasche Verbesserung des Hautbefundes (Abb. 1b) mit einer Reduktion des PASI von 34,2 auf 3,6 (Abb. 2c) sowie Rückgang des DLQI von 21 auf 3 (Abb. 2d).

## Diskussion

Eine HIV-Erkrankung kann sich auf unterschiedlichste Weise manifestieren, oftmals in Form von Infektionserkrankungen oder Malignomen. Kutane Manifestationen sind ebenfalls möglich wie das Neuauftreten oder die hier dargestellte Exazerbation einer bekannten Psoriasis vulgaris [3]. Die HIV-assoziierte Psoriasis geht häufig mit einem schweren und zum Teil therapieresistenten Verlauf sowie einem untypischen Hautbefund einher [8].

Die Psoriasis vulgaris gilt als T‑Zell-vermittelte Erkrankung mit einer Überexpression inflammatorischer Zytokine. Es erscheint auf den ersten Blick paradox, dass es im Rahmen einer Infektion, die durch eine abnehmende Immunantwort gekennzeichnet ist, zur Exazerbation der Psoriasis kommt. Eine mögliche Erklärung könnte die verminderte Anzahl regulatorischer T‑Zellen und ein Ungleichgewicht zwischen CD4^+^- und CD8^+^-Lymphozyten darstellen, was eine erhöhte Zytokinausschüttung und Verstärkung der autoimmunen Reaktivität zur Folge hat [2].

In der S3-Leitlinie der Psoriasis finden sich Empfehlungen für das therapeutische Vorgehen bei unterschiedlichen klinischen Konstellationen wie das Vorliegen einer Malignomerkrankung oder Infektionserkrankungen wie Virushepatitis und Tuberkulose [7]. Empfehlungen für die HIV-assoziierte Psoriasis sind aktuell nicht enthalten. Für die Behandlung dieser Patienten fehlen randomisierte kontrollierte Studien, sodass sich Therapieempfehlungen insbesondere auf Fallberichte stützen. Da ein Anstieg der CD4^+^-Lymphozyten häufig mit einer Verbesserung des Hautbefundes einhergeht, stellt die antiretrovirale Therapie nicht nur für die HIV-Infektion, sondern auch für die Psoriasis einen wichtigen Behandlungsbaustein dar. Während für gering ausgeprägte Formen der HIV-assoziierten Psoriasis eine additive topische Behandlung ausreichend ist, können schwere Formen mit einer Lichttherapie oder oralen Retinoiden behandelt werden. Für therapierefraktäre Verläufe oder bei begleitender Psoriasis-Arthritis kann die Anwendung klassischer Immunsuppressiva oder Biologika evaluiert werden [1]. Gemäß eines Reviews aus dem Jahr 2020 finden sich in der Literatur die meisten Fallberichte zum Einsatz von Biologika bei HIV-assoziierter Psoriasis für TNF-α-Inhibitoren und den Interleukin-12/23-Inhibitor Ustekinumab [4]. Auch wenn die Datenlage gering ist, könnten Biologika aufgrund positiver Effekte auf die CD4^+^-Lymphozyten und die HIV-Viruslast sowie eines geringeren Risikos opportunistischer Infektionen den konventionellen Systemtherapien wie Methotrexat überlegen sein [5]. Eine evidenzbasierte Empfehlung zur Systemtherapie bei HIV-assoziierter Psoriasis ist anhand der aktuellen Datenlage jedoch nicht möglich.

Gemäß der S3-Leitlinie der Psoriasis vulgaris wird eine HIV-Serologie im Rahmen der Voruntersuchungen vor Einleitung einer Biologikatherapie empfohlen [6]. In unserem Fall erwiesen sich diese Untersuchung und die hieraus folgende antiretrovirale Therapie als hocheffektiv zur Behandlung der HIV-assoziierten Psoriasis. Daher sollte bei Patienten mit plötzlicher Exazerbation oder Therapieresistenz – unabhängig von empfohlenen Voruntersuchungen für Systemtherapien – eine HIV-Infektion als möglicher Auslöser bedacht werden.

## Fazit für die Praxis

- Bei Patienten mit plötzlicher Exazerbation oder Therapieresistenz einer Psoriasis vulgaris sollte eine HIV-Infektion als potenzieller Auslöser bedacht werden.
- Die antiretrovirale Therapie stellt eine hocheffektive Behandlungsoption einer HIV-assoziierten Psoriasis dar.
- Eine evidenzbasierte Empfehlung zur Systemtherapie bei HIV-assoziierter Psoriasis ist anhand der aktuellen Datenlage nicht möglich.
